# Deciphering the role of interferon alpha signaling and microenvironment crosstalk in inflammatory breast cancer

**DOI:** 10.1186/s13058-019-1140-1

**Published:** 2019-05-06

**Authors:** Olivia K. Provance, Joan Lewis-Wambi

**Affiliations:** 1 Department of Cancer Biology, University of Kansas Medical Center, 3901 Rainbow Boulevard, Wahl Hall East 1031, Kansas City, KS 66160 USA; 2 The University of Kansas Cancer Center, 3901 Rainbow Boulevard, Kansas City, KS 66160 USA

**Keywords:** Inflammatory breast cancer, Interferon alpha, Interferon-stimulated genes, IFITM1, STAT, Dendritic cells, Macrophages, Fibroblasts, Endothelial cells

## Abstract

Inflammatory breast cancer (IBC) is the most rare and aggressive subtype of breast cancer characterized by clusters of tumor cells invading lymph vessels, high rates of metastasis, and resistance to systemic chemotherapy. While significant progress has been made in understanding IBC, survival among IBC patients is still only one half that among patients with non-IBC. A major limitation to the development of more specific and effective treatments for IBC is a lack of identifiable molecular alterations that are specific to IBC. Emerging evidence suggests that the aggressive nature of IBC is not specific to IBC cells but instead driven by the interplay between autonomous signaling and context-dependent cytokine networks from the surrounding tumor microenvironment. Recently, the type I interferon, specifically the interferon alpha signature, has been identified as a pathway upregulated in IBC but few studies have addressed its role. Activation of the interferon alpha signaling pathway has been shown to contribute to apoptosis and cellular senescence but is also attributed to increased migration and drug resistance depending on the interferon-stimulated genes transcribed. The mechanisms promoting the increase in interferon alpha expression and the role interferon alpha plays in IBC remain speculative. Current hypotheses suggest that immune and stromal cells in the local tumor microenvironment contribute to the interferon alpha signaling cascade within the tumor cell and that this activation may further alter the immune and stromal cells within the microenvironment. This review serves as an overview of the role of interferon alpha signaling in IBC. Ideally, future experiments should investigate the mechanistic interplay of interferons in IBC to develop more efficacious treatment strategies for IBC patients.

## Background

Inflammatory breast cancer (IBC) is the most aggressive and lethal subtype of breast cancer accounting for 3% of total breast cancer diagnoses but 10% of total breast cancer-related deaths [1]. As of 2018, the 5-year survival rate for IBC is 40–60% [2] and the 15-year survival rate is approximately 20% [3]. IBC presents with distinctive features that are absent in non-IBC allowing for a clinical diagnosis. These features include rapid redness and swelling of the breast, generalized induration, and peau d’orange skin consistency often accompanied by a pathological confirmation of invasive carcinoma through a skin-punch biopsy [2]. It is hypothesized that the clinical symptoms manifest due to blockage of dermal lymph vessels by aggregates of tumor, immune, and stromal cells termed tumor emboli, a hallmark of IBC [2]. Despite these differences, IBC is similar to non-IBC in terms of molecular subtyping. All breast tumors are characterized based on receptor status (estrogen receptor, ER; progesterone receptor, PR; human epidermal growth factor-2, HER2) and a panel of genes contributing to predictive and prognostic indices [4]. IBC is comprised of the same molecular subtypes as non-IBC: ER/PR+ (35–40%, 60–70% in non-IBC), HER2+ (35–50%, 20% in non-IBC) and triple-negative (TN) (20–40%, 15–20% in non-IBC) [5–7]. Though the molecular subtypes overlap, to date, IBC remains significantly more aggressive and there is no IBC-specific therapy. The current treatment involves a trimodality approach of neoadjuvant chemotherapy, mastectomy, and axillary lymph node removal followed by post mastectomy radiation [2]. Despite aggressive treatment, IBC patients face significantly higher incidence of metastatic disease compared to non-IBC; therefore, there is an unmet need to determine efficacious treatment strategies for IBC.

Researchers have turned to genetic profiling to identify molecular alterations distinct to IBC; however, these studies remain inconclusive perhaps due to the additional complexity driven by the tumor microenvironment (TME) [8]. Key components of the TME in IBC include dendritic cells, macrophages, fibroblasts, and endothelial cells. The presence of these cells within the microenvironment may contribute to the intrinsic factors of IBC. For example, cancer-associated fibroblasts are a key driver of breast cancer progression [9], macrophages mediate IBC migration [10, 11], and IBC tumors have a high infiltration of endothelial cells secreting angiogenic factors such as vascular endothelial growth factor (VEGF) and platelet-derived growth factor (PDGF) [8, 12]. Though studies have begun to elucidate the tumor-promoting interplay of these cell types, much research is still needed.

Recently, studies have suggested that IBC cells have increased levels of interferon alpha (IFNα) [13, 14]. Interferons are cytokines known for their ability to modulate the innate and adaptive immune system through both autocrine and paracrine signaling [15], providing an interesting and novel avenue for IBC microenvironment research. While direct analysis supporting a role for IFNα signaling in IBC is sparse, extrapolation of data from aggressive breast tumors provides insight into the role of IFNα in IBC. In this review, we summarize the pre-clinical findings supporting a role for IFNα in IBC progression, discuss the potential molecular mechanisms regulated by IFNα in IBC, and predict how the signaling interplay between the microenvironment and IFNα secreted by IBC tumor cells contribute to the pro-tumorigenic milieu. Though we recognize that IFNβ and IFNγ also play an important role in breast cancer progression, our review will focus solely on IFNα.

### Interferon alpha in inflammatory breast cancer

In 2014, Bertucci et al. [13] published a study with the largest cohort of non-treated IBC samples to date. DNA microarray expression profiling identified the IFNα pathway as being the only pathway that was significantly upregulated in IBC. Pre-clinical evidence supports this finding but suggests that IFNα upregulation is specific to TN-IBC [14]. In comparing the SUM149 and SUM190 cell lines (TN-IBC and HER2+ IBC, respectively), the mRNA and the secreted levels of IFNα are significantly higher in TN-IBC compared to HER2+ IBC [14]. Though interferons are known to confer an antiviral state to cells, evidence suggests that breast cancer tumors expressing high IFN response genes are 1.7 times more likely to metastasize as compared to tumors expressing low levels of these genes, providing pre-clinical evidence for a pro-tumor face of IFNα [16].

### An overview of interferon alpha biology and signaling

IFNα belongs to the family of type I interferons which also consists of IFNβ [17]. Thirteen subtypes of IFNα have been identified. Each member of IFNα lacks distinct introns and is encoded independently with genes located in a 400-kb cluster on chromosome 9q [18]. Notably, IFNα and IFNβ both signal through the interferon alpha receptor-(IFNAR)1 and IFNAR2 complex but have different binding affinities thus producing distinct antiviral effects [17]. Therefore, IFNα and IFNβ are not interchangeable.

**Fig. 1:**
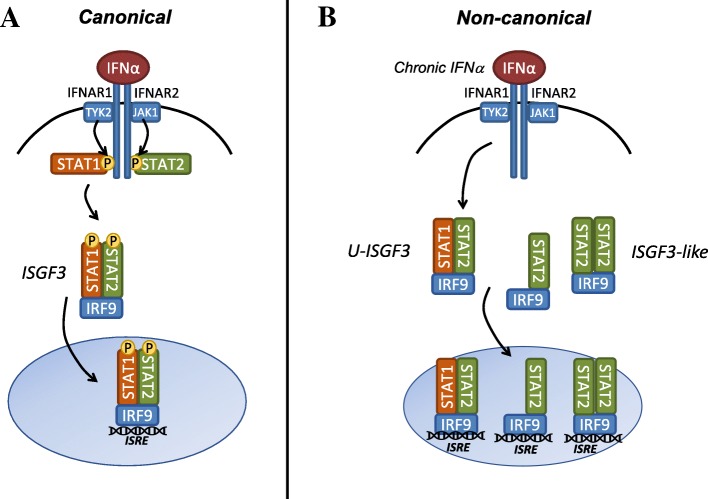
Downstream IFNα signaling in IBC. **a** Canonical IFNα signaling begins with IFNα binding to IFNAR1/IFNAR2 receptor. After it binds to the receptor, JAK1 and TYK2 cross-phosphorylate each other and phosphorylate the intracellular domains of the receptors to allow for STAT1 and STAT2 binding through their SH2 domain for subsequent phosphorylation. Once STAT dimers form, they translocate into the nucleus and bind to their respective DNA binding elements. IRF9 and STAT1 are the only two proteins that directly interact with the DNA whereas STAT2 is necessary for stabilizing the ISGF3 complex and further recruitment of co-activators. **b** During chronic IFNα signaling, STAT proteins are no longer robustly phosphorylated however the interferon signature may remain upregulated due to the similarity between ISGF3 and U-ISGF3. In the absence of IFNα, there will either be no signal or, depending on STAT2 and IRF9 levels, the interferon response may stay elevated due to the continued formation of STAT2 and IRF9. TN-IBC cells have robust levels of IRF9 and IRF9 increases in tumor clusters. Therefore, IRF9 could be the key driver of this response. Adapted from [19]

For interferons to elicit their effect, they must bind to their specific receptor which is outlined in Fig. 1a (Adapted from [19]). IFNα binds to IFNAR1 stimulating a conformational change and recruitment of IFNAR2 to dimerize. IFNAR1 and IFNAR2 do not have intrinsic kinase activity but are constitutively associated with Janus-activated kinase-1 (JAK1) and tyrosine kinase-2 (TYK2) [20]. Once IFNα binds to IFNAR1, a conformational change occurs and IFNAR2 dimerizes bringing JAK1 and TYK2 into close proximity with one another promoting cross phosphorylation of JAK1 and TYK2 and subsequent phosphorylation of the intracellular domain of IFNAR1 and IFNAR2 [20]. Phosphorylation of the IFNAR receptors provides a receptor-docking site for signal transducers and activators of transcription (STAT)-1 and STAT2. STAT1 and STAT2 are recruited to the receptors through their SH2 domain thus allowing them to be phosphorylated by JAK1 and TYK2. Phosphorylated STATs may then homodimerize or heterodimerize. Homodimers of STAT1 are known as IFNα-activated factor (AAF) whereas heterodimers of STAT1/STAT2 associated with interferon regulatory factor (IRF)-9 is termed interferon-stimulated gene factor-3 (ISGF3). These complexes translocate into the nucleus and bind to gamma-activated sequences (GAS) or interferon-stimulated response elements (ISREs) in DNA, respectively, transcribing interferon-stimulated genes (ISGs) which are listed in Table 1 (adapted from [21–23]).

**Table 1:** Transcription factor complexes and their control of interferon-stimulated genes

ISGF3		U-ISGF3	GAS
MX1/2	IRF9	MX1/2	IFITM1
IFITM1	IRF4	IFITM1	STAT4
PLSCR1	cGAS	PLSCR1	IRF1
OAS1/2/3	STAT1	OAS1/2/3	IRF8
SOCS	STAT2	IRF7	IDO
USP18	NOS2	STAT1	NOS2
ISG15		IFI27	PDGFRA
IRF3		IFI44	KRT14
IRF7			IRF2

*ISGF3* (interferon-stimulated gene factor 3), consists of phosphorylated STAT1 and STAT2 dimers bound to IRF9; *U-ISGF3* (unphosphorylated interferon-stimulated gene factor 3), consists of a dimer of unphosphorylated STAT proteins bound to IRF9; *GAS* (gamma activated sequence), consists of phosphorylated STAT1 dimers

Gene transcription mediated by IFNα is tightly regulated by multiple processes including stability of the IFNα/IFNAR1/IFNAR2 complex, the concentration of IFNAR1/2 on the membrane and transcription of negative regulators including suppressor of cytokine signaling (SOCS) and ubiquitin-specific peptidase (USP) 18 [17]. An in-depth discussion of these regulatory processes and how it pertains to IBC is beyond the scope of this review but has been previously discussed [17]. Regardless, these factors are important to consider for future studies on IFNα signaling in IBC.

### Intracellular regulation of interferon alpha production

#### Transcriptional regulation of interferon alpha

**Fig. 2:**
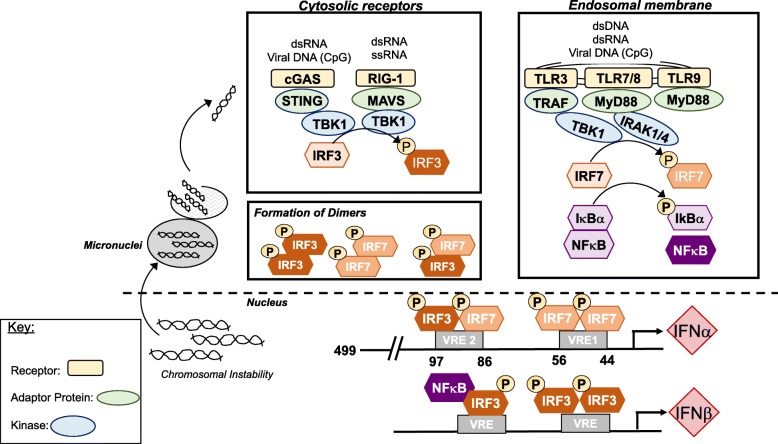
Transcriptional activation of interferon alpha and beta: Type 1 interferon signaling may be stimulated by either cytosolic receptors or endosomal membrane receptors in the presence of double-stranded RNA (dsRNA), viral DNA, single-stranded RNA (ssRNA), or double-stranded DNA (dsDNA). Specific adaptor proteins bridge the receptor and the respective kinase. Upon kinase activation, IRF3 and IRF7 are phosphorylated by specific kinases and NFκB can be released from its inhibitory complex and translocate into the nucleus. Once IRF3 and IRF7 are phosphorylated, they form dimers. Dimerization partners are determined based on the site of phosphorylation and the levels of each protein. Once dimers form, they translocate into the nucleus and bind to the respective responsive element. IFNα has two viral response elements 30 kb apart (VRE1 and VRE2). VRE1 has preferential for IRF7 dimers whereas VRE2 has preferential binding for IRF3/IRF7 dimers but both are not necessary for full transcription [26]. IFNβ is preferentially transcribed by IRF3 and NFκB. IFNβ can further promote the activation of IFNα through stimulating the IFNAR receptor which produces increased levels of IRF7 (Fig. 1)

Stimulation of IFNα transcription is a complex process and is outlined in Fig. 2. Canonically, production of IFNα is driven by activation of endosomal membrane localized toll-like receptors (TLRs) or cytoplasmic localized retinoic acid-inducible gene I (RIG1) receptors and cyclic GMP-AMP synthase (cGAS) receptor by combinations of double-stranded DNA (dsDNA), double-stranded RNA (dsRNA), single-stranded RNA (ssRNA), or by viral DNA [24, 25]. Adaptor molecules associated with the receptors then stimulate specific kinase activity promoting phosphorylation of interferon regulatory factor (IRF3) and IRF7 or degradation of IκB for NFκB translocation into the nucleus [22]. Importantly, each IFNα family member is transcribed by IRF3 or IRF7; however, the binding sites for these transcription factors are slightly altered in each gene [26]. Since the majority of what is known about IFNα transcription is derived from studies focusing on viral replication, we will discuss the potential regulatory mechanisms promoting increased IFNα levels in IBC through extrapolating data from viral activation and breast cancer in general and applying it to IBC.

#### Mechanisms contributing to interferon alpha transcription in IBC

In the absence of bacterial or pathogenic signals, cancer cells can transcribe IFNα in response to endogenous danger signals driven by persistent DNA damage response because of chromosomal instability (CIN) [27] (Fig. 2). Cancer cells have a high error rate of chromosome segregation during mitosis thus leading to CIN which results in ruptured micronuclei releasing genomic DNA into the cytosol triggering an interferon response through sensing by cGAS-STING and subsequent phosphorylation of IRF3 [27, 28] (Fig. 2). A study analyzed CIN in a microarray dataset of lymph node-negative primary breast cancer patients prior to systemic therapy and found that ER-negative, triple-negative, and basal-like tumors have significantly higher CIN scores [29] which is correlated with tumor metastasis [27]. Further supporting this data, a DNA microarray showed that compared to non-IBC samples, IBC had higher levels of TLRs, increasing the sensitivity of DNA damage response detection [5]. Therefore, IBC may be prone to an IFNα gene signature due to high levels of CIN but this remains to be tested.

Though there is limited information regarding IFNα in IBC, it is widely accepted that NFκB is elevated in IBC cell lines and patient tumors. IFNα could perhaps be controlled via NFκB indirectly since NFκB transcribes IFNβ which can promote transcription of IRF7 and IRF3, key regulators of IFNα [30]. Interestingly, it has been observed that the CD95/FADD apoptotic pathway regulates type I interferon production through stimulation of the NFκB axis thus leading to stemness, an intrinsic phenotype of IBC [31]. Stemness combines the ability of a cell to perpetuate its lineage, to give rise to differentiated cells and to interact with its environment to maintain a balance between quiescence, proliferation, and regeneration. Cancer stem cells (CSCs) display stemness in various circumstances, including the sustaining of cancer progression and the interaction with their environment in search for key survival factors. As a result, CSCs can recurrently persist after therapy which is probably a major contributing factor to the high rates of relapse observed for IBC.

Moving forward, it is important to consider investigating the genetic amplification of factors regulating IFNα signaling, post transcriptional modifications of these factors, and the intricate web of feedback and feedforward mechanisms that may be contributing to increased IFNα.

### Interferon alpha-stimulated genes in IBC tumors and resulting phenotypes

We have established that IFNα may contribute to IBC tumor aggression; however, the specific role of IFNα is determined by many factors including its concentration, the specific tumor type, and the duration of the stimulus. For example, induction of antiviral activity requires a few hours of IFNα exposure whereas antiproliferative activity requires constant exposure or high concentrations of IFNα [32]. IFNα has been used as a potential therapeutic agent due to its ability to induce potent antiproliferative and even apoptotic cellular responses used at high concentrations, but concentrations of IFNα necessary for induction of apoptosis are a thousandfold higher than normally produced IFNα [32]. Therefore, ISGs produced at lower concentrations could be detrimental to the host by contributing to IBC cell tumorigenicity. IFNα can control proliferation, differentiation, survival, invasion, drug resistance, and radioresistance in tumors due to JAK/STAT activation and ISG expression [33]. Notably, these phenotypes may be present if JAK/STAT signaling is not fully active.

#### Non-canonical interferon stimulated gene induction by interferon alpha in IBC

STATs can promote their effect independent of phosphorylation from canonical IFNα signaling as outlined in Fig. 1b. Canonically, during an interferon response, STATs are initially phosphorylated and IFNα signaling is initiated and resolved quickly, but during chronic inflammation STATs lose their phosphorylation status even in the presence of IFNα contributing to prolonged antiviral effects [21]. These stats are known as unphosphorylated STATs (U-STATS). A U-STAT1/U-STAT2 dimer with IRF9 forms an active, unphosphorylated ISGF3 (U-ISGF3) complex (Fig. 1b). In the presence of IFN, high levels of IRF9 may switch the balance from ISGF3-mediated transcription to U-ISGF3 signaling [34]. Importantly, ISGs stimulated by ISGF3 and by U-ISGF3 differ (Table 1), and we have found that TN-IBC cells lack basal levels of phosphorylated STAT1 and STAT2 in culture [14]. Aside from U-ISGF3 signaling, an ISGF3-like complex can form between STAT2 and IRF9 which can transcribe ISGs independently of STAT1 [19]. However, in these same cells, treatment with a low dose of exogenous IFNα promotes initial phospho-STAT activity but these levels decrease overtime while the expression of an ISG termed interferon-induced transmembrane protein-1 (IFITM1) production is markedly increased in TN-IBC cells [14]. This suggests that the IFNα pathway is not saturated at basal levels in TN-IBC indicating three things: (1) TN-IBC may still be able to respond to exogenous IFN treatment in both a pro- and anti-tumor manner depending on concentration; (2) this pathway may be stimulated on a basal level to confer autocrine signaling to the tumor, promoting increased aggression by mediating the levels of STAT1, STAT2, and IRF9; or (3) TN-IBC cells may not be sensitive to autocrine IFNα signaling. Therefore, depending on the quantity and the length of IFNα stimulus, different ISGs may be transcribed resulting in cellular phenotypic differences which may be specific to IBC as a whole or within IBC subtypes.

Aside from non-canonical IFNα signaling, IFNα is known to mediate activation of other cellular pathways through intracellular crosstalk. Interestingly, a mechanism whereby IFNα may lead to cellular aggression instead of apoptosis is through activation of NFκB signaling via STAT3 and PI3K/AKT [35]. This pathway is independent of the ISRE-driven ISG gene expression, and not all ISGs have NFκB binding sites. Future studies elucidating these relationships may provide insight into how to target TN-IBC since NFκB has been shown to be elevated in IBC. Previous studies investigating autocrine IFNα signaling crosstalk are sparse and focus mainly on cellular responses to exogenous IFNα treatment. Studies have suggested IFNα can modulate PDGF and TGFβ signaling which could provide insight into autocrine IFNα signaling under the caveat that cellular responses to IFN are dose and time dependent [36]. The plasticity of the cellular response to IFNα has been extensively reviewed by Cheon et al. [37] and Medrano et al. [32]*.*

Emerging evidence suggests that ISGs are responsible for the aggressive phenotype of many cancers. A few of which are outlined in Table 2 and how they are implicated in IBC [14, 17, 22, 38]. An emerging player in TN-IBC is IFITM1. IFITM1 is a 17-kDa transmembrane protein located on 11p15 and can be transcribed in all cell types. IFITM1 is well known as a mediator of viral entry and replication and has recently gained popularity in cancer. IFITM1 may be induced by ISGF3 or U-ISGF3 making this a non-canonical ISG. We have previously identified IFITM1 as an ISG overexpressed in TN-IBC compared to HER2+ IBC [14]. Inhibiting IFITM1 expression in TN-IBC attenuates the proliferation and migration of these cells [14]. Other studies in aromatase inhibitor-resistant breast cancer [39],colorectal cancer [40],  gliomas, [41], and head and neck cancer [42] have reported similar function of IFITM1. Furthermore, IFITM1 has been implicated in the radioresistant phenotype in head and neck, breast, prostate, lung, and brain cancers as discussed by Weichselbaum et al. [38].

**Table 2:** Interferon-stimulated genes implicated in IBC and their specific functions

Function	Function in IBC	ISG	Reference
DNA damage resistance	Inferred	STAT1, IFI27, MX1/2, PLSCR1, OAS1/2/3, IRF9, IFITM1	[14, 38]
Migration/invasion	Confirmed	IFITM1, STAT2	[14]
Positive feedback	Inferred	IRF3, IRF7, IRF9	[22]
Negative regulation	Inferred	SOCS1, USP18	[17, 22]

A selected list of ISGs stimulated by IFNα is listed with corresponding function derived from the literature. The confirmation of the function of ISGs in IBC is outlined. All ISGs have confirmed expression in IBC in our unpublished data

Though STATs are not active, low levels of IFNα may be sustaining ISG production. Perhaps this is due to IFN control on STATs themselves. We have previously shown that siRNA against IFNα decreases STAT2 levels but not STAT1 levels in TN-IBC. Additionally, loss of STAT2, but not STAT1, was associated with decreased proliferation and migration in TN-IBC cells [14]. Therefore, stabilization of the U-ISGF3 complex could be a potential mechanism whereby TN-IBC utilizes IFNα for aggression. Future studies are needed to determine if and what ISGs contribute a feedback mechanism for IFNα as well as how these signaling mechanisms are altered in the presence of exogenous IFNα.

### Interferon alpha in the IBC tumor microenvironment

IFNα production from the tumor cells may have the capability of educating the microenvironment to be pro-survival for tumor cells. Likewise, tumor microenvironment (TME) components may be able to promote or enhance the migratory and invasive capacities of IBC cells, perhaps through influencing JAK/STAT signaling. Interestingly, one study has shown that 63.3% of breast cancers are positive for IFNα and that stage III breast cancers had no IFNα binding to the receptor complex suggesting constitutive secretion of IFNα but a lack of autocrine signaling [43]. JAK/STAT signaling is known to be both a cell intrinsic and cell extrinsic mechanism of IBC aggression [8], but the molecular mechanisms of this reciprocal signaling remain to be elucidated. If this mechanism is understood, perhaps a therapy targeting both the tumor microenvironment and tumor cells can be developed.

**Fig. 3:**
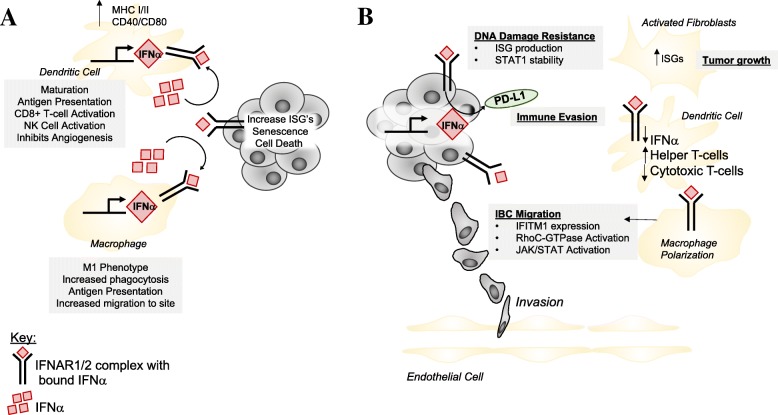
Proposed implications of IFNα signaling between IBC tumor and the tumor microenvironment. **a** During canonical IFNα signaling which often occurs during the first steps of immunosurveillance, dendritic cells and macrophages promote tumor cell killing through release of high levels of IFNα. Dendritic cells have high levels of MHC class I and II as well as co-activators (CD40/CD80) which promote the cytotoxic T cell response against the tumor. Macrophages are often polarized to be more M1-like in this scenario. IFNα promotes increased migration of M1-macrophages to the inflamed tissue to aid in antigen presentation. **b** Hypothesized alterations due to chronic IFNα signaling. IFNα secreted from the tumor cells may act in an autocrine or paracrine manner. In an autocrine fashion, IFNα binds to the receptors on IBC tumor cells increasing PD-L1 expression and upregulating canonical and non-canonical JAK/STAT signaling. Immune evasion may occur through release of IFNα and the paracrine effect on dendritic cells. We hypothesize that increased levels of IFNα produced in the tumor and secreted into the TME in a chronic manner essentially exhausts dendritic cells inhibiting their capability of simulating cytotoxic T cells while instead the helper T cell population is expanded. Furthermore, regulation of JAK/STAT signaling by IFNα may increase DNA damage resistance through increasing ISG production. Additionally, IBC migration is altered through interaction with macrophages. Tumor-associated macrophages in IBC are shown to enhance RhoC-GTPase activation and JAK/STAT signaling by macrophages makes these cells highly aggressive due to their invasion into the blood vessels and the high rate of angiogenesis in IBC. Fibroblasts and endothelial cells in IBC remain understudied; however, macrophages may have the capability to increase the activation of these cells. Adapted from Lim et al. [8]

In this section, we discuss the role of IFNα on immunosurveillance and how this may lead to neoplastic initiation and IBC progression followed by a discussion of how IFNα may impact different cell types within the IBC TME. Figure 3a outlines how initial immunosurveillance driven by IFNα may promote a cytotoxic response against the tumor, and Fig. 3b shows how IFNα from the tumor cell may promote an altered, pro-tumorigenic immune milieu specific to IBC.

#### The contribution of interferon alpha to tumor cell immune escape: current mechanistic hypotheses

As previously discussed, IBC tumor emboli consist of tumor cells and immune cells and are highly metastatic [2, 8]. Therefore, we hypothesize that IFNα signaling may support the survival of cancer cells and their escape from detection in immune cells.

During initial tumor formation, immune cells surrounding the tumor will recognize tumor-specific antigens to eliminate the transformed cells. Canonically, IFNα is a known tumor suppressor by inducing cellular senescence and death through recruiting dendritic cells and activating T cells [44]. However, a review published by Coppe et al. [45] introduces the senescence-associated secretory phenotype (SASP) that may be altered due to chronic inflammation. Normal cells undergoing the DNA damage response increase secretion of inflammatory cytokines which may halt their division. During senescence, normal epithelial cells become transformed through destabilization of chromatin, increased DNA damage, or through mutations necessary for survival. The secretory phenotype of transformed cells then promotes a pro-tumorigenic microenvironment through immune suppression; therefore, production and release of IFNα may contribute to immune suppression by upregulating the levels of PD-L1 on the tumor cell [46] (Fig. 3b).

The mechanism by which immune and stromal cells may drive the upregulation of tumor secreted IFNα remains elusive. Perhaps the stroma releases paracrine signals to neoplastic cells which then maintain these signals through endogenous autocrine signaling, promoting transformation and allowing cancer to progress with an ever-changing microenvironment. Regarding this crosstalk, we hypothesize that low levels of IFNα produced from immune cells in the microenvironment promote the transformation of epithelial cells with an increase in IFNα which subsequently makes the microenvironment conducive to tumor progression. This has been observed through chronic treatment with IFNβ, showing that specific ISGs remain activated in the absence of IFNβ as well as during chronic interferon signaling [21]. Moving forward, we will discuss how dendritic cells, tumor-associated macrophages, cancer-associated fibroblasts, and endothelial cells may contribute and respond to elevated IFNα from IBC tumor cells, promoting a pro-tumorigenic TME milieu. For more information refer to Cheon et al. [37] and Medrano et al. [32] for a comprehensive review of the TME and type-1 interferons.

#### Dendritic cells

Dendritic cells (DCs) are activated by and are key producers of IFNα [47]. There are two subgroups of dendritic cells, plasmacytoid-derived DCs (pDCs), and myeloid-derived DCs (mDCs); both are known for transformed cell elimination [48]. In normal DC activation by IFNα, DCs increase their levels of MHC class I and II, as well as T cell co-activators (CD40, CD80, CD86 and CD83) thus promoting a cytotoxic T cell response (Fig. 3a) [48]. Contrary to their canonical role, accumulation of mature dendritic cells has been correlated with increased metastasis in breast cancer [49]. Mature DCs are IFNα deficient thus promoting expansion of regulatory T cells associated with immune tolerance and poor clinical outcome [50]. This is in line with the current knowledge about DCs in IBC such that activated DCs are decreased in IBC [8]. Therefore, IFNα is an important mediator of dendritic cell activation and subsequent T cell response in the tumor microenvironment. We speculate that IBC secretion of IFNα essentially exhausts dendritic cells and T cells, therefore inhibiting the cytotoxic response (Fig. 3b).

#### Tumor-associated macrophages

Macrophages are the most abundant leukocyte present in the tumor stroma and display a high level of plasticity allowing adaptation to environmental stimuli. Despite popular evidence, macrophages do not exist as strictly M1 (anti-tumor) or M2 (pro-tumor) phenotypes, but instead lie within the spectrum. M1 polarization occurs due to exposure to interferon (IFN) γ, lipopolysaccharides (LPS), or TNFα produced by Th1, natural killer, or antigen presenting cells [51]. M1-polarized macrophages facilitate tumoricidal responses and inflammation [52]. M2 polarization is stimulated by IL-4, IL-13, and IL-10, or stress hormones, have poor antigen presenting capability, suppress T cell activation, and only exhibit minor cytotoxicity for tumor cells due to their limited ability to produce nitric oxide and pro-inflammatory cytokines [52].

IFNα is known to promote macrophages to favor the M1 phenotype (Fig. 3a) [53]; however, clinical and pre-clinical evidence in aggressive breast tumors and IBC seems to contradict this idea. Allaoui et al. [54] compared luminal, ER+ non-IBC, tumors and triple-negative non-IBC breast cancer tumors and found that the level of M2-like macrophages is significantly increased in triple-negative tumors. An in vitro study with SUM149 cells and THP-1 monocytes showed that during co-culture conditions; SUM149 cells promoted the immune suppressive phenotype of THP-1 monocytes [55]. Macrophages have further been shown to promote migration via RhoC-GTPase signaling in IBC (Fig. 3b) [11].

Perhaps IFNα secreted from the IBC tumor cells at a low dose avoids the rapid induction of M1 polarization while the chronic production eventually alters the macrophage phenotype, increasing the complexity of this relationship. Evidence suggests that macrophages are a key player in the microenvironment in IBC, yet many questions remain to be addressed. Specifically, how is IFNα a key regulator of macrophages within the IBC tumor TME and what are the other macrophage cell-cell relationships that are altered.

#### Cancer-associated fibroblasts

Cancer-associated fibroblasts (CAF’s) are stromal cells that synthesize ECM and collagen and aid in wound healing. Though the involvement of CAFs in IBC is understudied, it is known that CAFs with high levels of IFNα response genes promote the growth of breast cancer MCF-7 (ER+, non-IBC) cells in an in vitro co-culture method (Fig. 3b) [56]. Though signaling processes are different between ER+ non-IBC and IBC breast cancer cells, this data provides insight into the importance of CAFs in breast cancer progression. Moreover, it has been shown that in aggressive breast cancer CAF levels are increased and that these activated CAFs can in turn regulate recruitment of macrophages [54]. Though IFNα may not have any known effects on CAFs in IBC, it can be postulated that the interplay between immune and stromal cells on the basis of the IFNα axis promotes IBC tumor aggression. Therefore, it is necessary to understand the effect of IFNα from IBC cells on the role of CAFs both in vitro and in vivo*.*

#### Endothelial cells

It is known that type I interferons normally have anti-angiogenic properties [57]. However, complex crosstalk between immune cells and stromal cells regulate tumor vasculature [32, 58]. IBC has elevated rates of infiltrating lymphatic endothelial cells compared to non-IBC [12]. Though this is regulated by VEGF signaling, the specific biological mechanisms promoting this are unknown. Perhaps the tumor cells do not directly act on the endothelial cells via IFNα signaling, but instead the interplay between the other immune and stromal cells previously discussed promotes increased infiltration. The direct relationship between IFNα, IBC, and endothelial cells remains to be determined.

## Conclusion

Evidence presented above suggests that IFNα is an important factor in IBC tumor development and progression but the functions of IFNα and how this cytokine may relate to other cellular processes remain unclear. IFNα signaling is comprised of multiple feedback loops, alternative signaling mechanisms, and communication between nearby cells contributing to multiple layers of system complexity. In IBC specifically, emerging evidence suggests that the tumor microenvironment is a key driver of onset and aggression. All cells can respond to IFNα and the response is dependent on concentration and tissue type making IFNα a highly pleiotropic cytokine.

IFNα as therapy has been extensively studied; however, targeting tumor produced IFNα or downstream signaling is new territory. Currently, ruxolitinib, a JAK1 and JAK2 inhibitor, is being investigated in treatment of primary and metastatic triple-negative breast cancer [59]. Ruxolitinib was well tolerated and, as expected, decreased JAK-STAT target genes and pSTAT3 activity; however, the primary efficacy endpoint was not met. The authors introduced the idea of intratumoral heterogeneity contributing to resistance to ruxolitinib. Perhaps, this could be due to alternative signaling mechanisms as outlined in Fig. 1b. Supporting our discussion of the importance of the tumor microenvironment in influencing IFNα signaling, these authors provide evidence for altered immune profiles based on tumor pSTAT3 and JAK2 expression. In addition to ruxolitinib, PD-L1 targeting therapies have emerged as a promising target for solid tumors including IBC and it has been discussed that IFNα can modulate tumor expression of PD-L1 [37]. Ultimately, the knowledge obtained by continued investigation of the role of type I interferon signaling in IBC not only sheds light on the molecular underpinnings of the disease but also provides novel mechanisms of targeted therapy and drug resistance to be considered in the context of translational medicine.

In future efforts to uncover the mechanistic insight into how IBC cells utilize IFNα for tumor progression, we must consider both intracellular signaling crosstalk and the tumor microenvironment. Doing so will help define the complexity of this system in hopes of contributing to the development of more efficacious therapies targeting both the tumor cell and the surrounding microenvironment.

## Abbreviations

AAF: IFNα-activated factor
AKT: Protein kinase B
CAF: Cancer-associated fibroblast
cGAS-STING: Cyclic GMP-AMP synthase-stimulator of interferon genes
CIN: Chromosomal instability
CSC: Cancer stem cell
DC: Dendritic cell
dsDNA: Double-stranded DNA
dsRNA: Double-stranded RNA
ER: Estrogen receptor
FADD: Fas-associated death domain
HER2: Human epidermal growth factor 2
IBC: Inflammatory breast cancer
IFITM1: Interferon-induced transmembrane protein-1
IFN: Interferon
IFNA: Interferon alpha (gene)
IFNAR1: Interferon alpha receptor 1
IFNAR2: Interferon alpha receptor 2
IFNGR: Interferon gamma receptor
IFNα: Interferon alpha
IFNβ: Interferon beta
IFNγ: Interferon gamma
IL10: Interleukin-10
IL13: Interleukin-13
IL4: Interleukin-4
IRAK: Interleukin-1 receptor associated kinase
IRF: Interferon regulatory factor
ISG: Interferon-stimulated gene
ISG15: Interferon-stimulated gene-15
ISGF3: Interferon-stimulated gene factor 3
IκB: NFκB inhibitor
JAK: Janus-activated kinase
LPS: Lipopolysaccharide
MAVS: Mitochondrial antiviral signaling protein
mDC: Myeloid-derived DC
MHC: Major histocompatibility complex
MYD88: Myeloid differentiation primary response gene
NFκB: Nuclear factor kappa B
pCR: Pathological complete response
pDC: Plasmacytoid-derived DC
PDGF: Platelet-derived growth factor
PR: Progesterone receptor
RIG: Retinoic acid-inducible gene
shRNA: Short hairpin RNA
siRNA: Short interfering RNA
SOCS: Suppressor of cytokine signaling
ssRNA: Single-stranded RNA
STAT1: Signal transducer and activator of transcription-1
STAT2: Signal transducer and activator of transcription-2
STING: Stimulator of interferon genes
TAM: Tumor-associated macrophage
TBK: TANK-binding kinase
TLR: Toll-like receptor
TME: Tumor microenvironment
TN: Triple-negative
TNFα: Tumor necrosis factor alpha
TN-IBC: Triple-negative inflammatory breast cancer
T-reg: Regulatory T cells, CD4+
TYK2: Tyrosine kinase 2
U-ISGF3: Unphosphorylated interferon-stimulated gene factor 3
USP18: Ubiquitin-specific peptidase 18
VEGF: Vascular endothelial growth factor
